# Testosterone Therapy and Associated Rates of Tendon Tear and Surgical Repair: A Retrospective Analysis

**DOI:** 10.1177/23259671261430731

**Published:** 2026-04-20

**Authors:** Al-Hassan Dajani, Timothy P. Liu, Guillermo Araujo-Espinoza, Kevin Muy, Seth Cope, Kristofer Jones, Thomas J. Kremen

**Affiliations:** † Department of Orthopaedic Surgery, University of California, Los Angeles, California, USA; Investigation performed at the University of California, Los Angeles, California, USA

**Keywords:** rotator cuff repair, rotator cuff tear, tendon rupture, testosterone

## Abstract

**Background:**

Testosterone replacement therapy (TRT) use is increasing in both men and women with demonstrated benefits for muscle strength, sexual function, and well-being. However, previous studies have linked exogenous testosterone to elevated rates of tendon injury in upper and lower extremities. TRT has also been associated with higher rates of surgical repair and markedly elevated reoperation rates. Limited institutional data are available on tendon rupture rates and treatment trends.

**Purpose:**

To investigate the association between prescription TRT and tendon rupture risk, with a specific focus on rotator cuff tear (RCT) incidence and repair/revision rates at a single academic institution.

**Study Design:**

Cohort study; Level of evidence, 3.

**Methods:**

We queried 1 institution's electronic health records using International Classification of Diseases (ICD-10) codes for patients ≥18 years with tendon rupture and prescription TRT use within 90 days of injury between 2015 and 2023. Individuals with risk factors predisposing them to tendon injury were excluded. A subanalysis of RCT was performed using a chart review of a subgroup of TRT patients and propensity-matched controls. Outcomes of interest included rupture location and rate, and, for the RCT, tear severity and rates of surgical repair/revision. Outcomes were compared between TRT users and nonusers.

**Results:**

We identified 410 TRT users and 14,474 nonusers with tendon rupture. Men on TRT had significantly higher rupture rates (3.6% vs 1.3%; odds ratio [OR], 2.88 [95% CI, 2.59-3.20]) across all ages and races. No significant increase in tendon rupture rate was observed in women on TRT. RCTs were the most common injury (77%). In the RCT subanalysis (78 TRT users, 355 matched controls), TRT users underwent nonoperative intervention more frequently than nonusers (74.4% vs 46.7%; OR, 3.3 [95% CI, 1.91-5.72]). Tear severity and revision rates did not differ significantly between groups.

**Conclusion:**

TRT is associated with increased tendon rupture risk in men but not women, potentially due to sex-specific differences in dosing. TRT users with RCT were less likely to undergo arthroscopic repair compared with matched controls, despite similar distributions of tear severity.

Testosterone replacement therapy (TRT) is becoming increasingly common, with a 27% increase in prescriptions and >400,000 new users in the United States^16^ between 2018 and 2022. TRT in men helps to replenish diminished circulating testosterone levels in aging individuals^9^ and has been demonstrated to improve muscle mass, strength, sexual function, and a general sense of well-being.^3^ In women, TRT is primarily used for treatment of hypoactive sexual desire disorder and is given at much lower doses^4,21^ to approximate physiological premenopausal levels.^5^ There is also growing interest in testosterone as a potential therapy for osteoporosis in women, and higher circulating testosterone may be associated with improved bone mineral density.^23,24^ While prescription testosterone is being used by providers to treat medical disorders in men and women, it is important to note that nonprescription use of testosterone and its synthetic analogs is also common. The lifetime prevalence of use is 3.3% globally, with 6.4% in men and 1.6% in women.^18^ Nonprescription use of testosterone and its synthetic analogs is often used for cosmetic enhancement to increase muscle mass and improve sports performance.^1^ The current ubiquity of and access to testosterone make this an important topic to study, especially when trying to understand the benefits and deleterious effects on users.

For example, TRT has been associated with musculoskeletal complications. Exogenous testosterone puts individuals at a higher risk for tendon injury and rupture due to an imbalance between muscle hypertrophy and tendon strength^7^ as well as direct alterations of tendon architecture with collagen fibril dysplasia.^10,13,22^ National database studies show that TRT is associated with increased rates of injury in the Achilles, distal biceps, quadriceps, and rotator cuff tendons.^2,12,15,19,20^ This elevated risk is also reported to be similar between men and women despite lower testosterone doses used for female patients.^19^ Notably, patients on TRT are reported to undergo higher rates of surgical rotator cuff repair (RCR), despite a greater than 20-fold elevated risk of repair failure requiring reoperation.^19^ It is unclear what surgeon- and patient-specific factors lead to increased operation rates in this high-risk patient population. Also, there is a lack of studies within institutional cohorts to provide greater granularity on patterns of care.

Therefore, this study aimed to build upon previous database reports and investigate the rate of tendon ruptures in patients on prescription TRT at a single institution from 2015 to 2023. We also characterized the rates of RCT and subsequent surgical repair and revision. We hypothesized that prescription TRT increases the risk of tendon rupture in men but not women due to significantly lower doses used in women.

## Methods

### Testosterone Therapy and Upper and Lower Extremity Tendon Rupture Rate

We conducted a retrospective analysis using the Informatics for Integrating Biology & the Bedside (i2B2) tool, running queries with International Classification of Diseases (ICD-10) codes for de-identified patient data from October 2015 to July 2023 at a single academic institution. The inclusion criteria included patients aged ≥18 years, diagnosed with a tendon rupture, and who had used or not used exogenous prescription testosterone or testosterone derivatives for at least 90 days before tendon rupture. The exclusion criteria included confounding factors that may predispose to tendon injury—including any history of smoking, use of potential tendon-weakening drugs within a 90-day window before rupture (eg, quinolones, glucocorticoids, antilipemic agents, and chemotherapy); diabetes; and inflammatory conditions (eg, systemic connective disorders, rheumatoid arthritis, vasculitides, inflammatory bowel disease, and diagnosis of malignant breast neoplasm). A full table for the inclusion and exclusion criteria is provided in Supplemental Table 1. We obtained descriptive data (including sex, age, and race) and outcomes (including rate and location of tendon rupture) using ICD-10 codes.

### RCT and Repair Subanalysis

**Figure 1.**
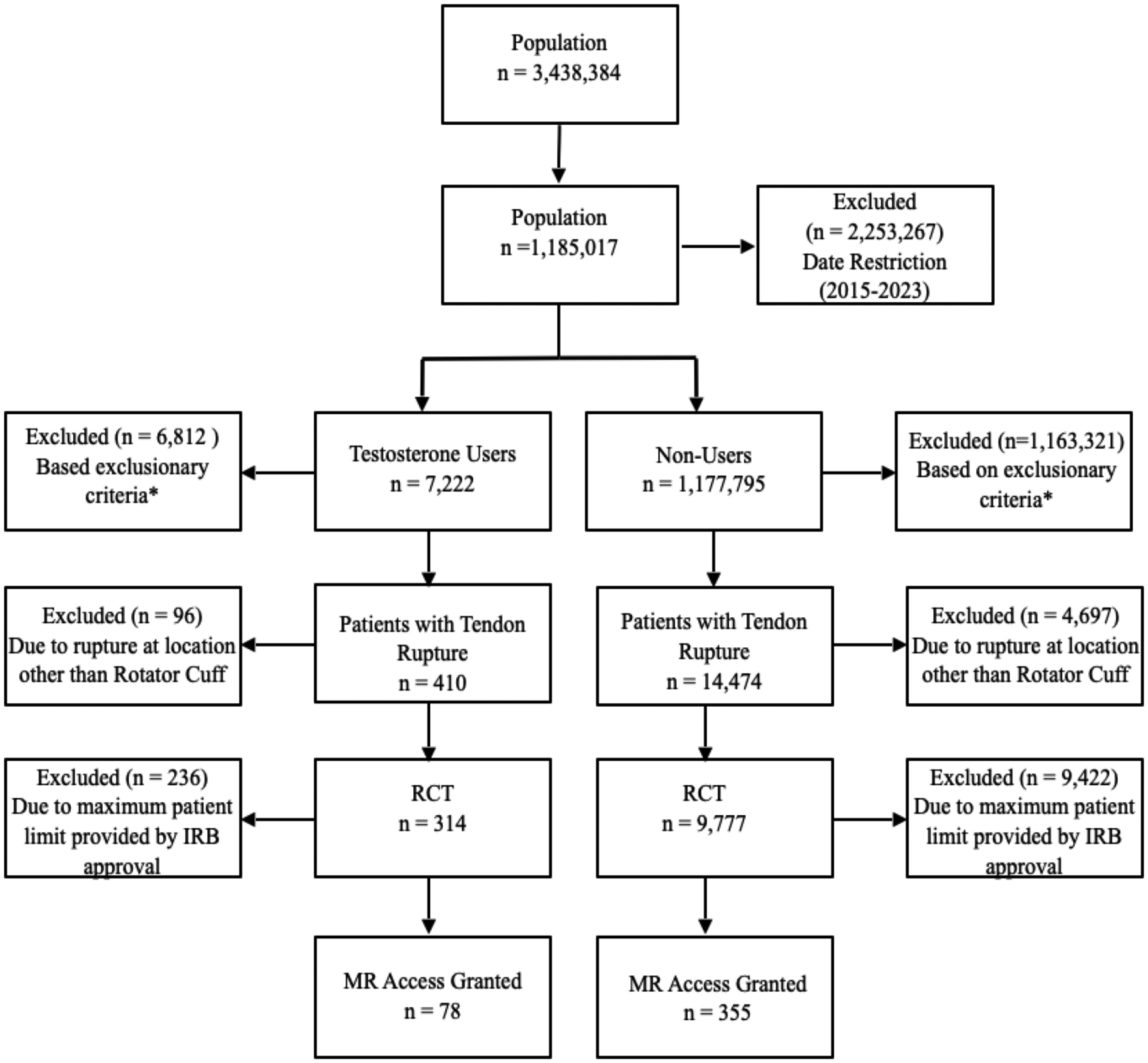
Flow diagram demonstrating exclusionary criteria and selection of patients of interest for analysis of tendon tears in patients on TRT and nonusers. The asterisk indicates specific exclusion and inclusion codes, which are detailed in Supplementary Table 1. IRB, institutional review board; MR, medical record; RCT, rotator cuff tear; TRT, testosterone replacement therapy.

To examine RCT trends and treatment in greater detail, we conducted an additional subanalysis using ICD-10 codes through our institution's Clinical and Translational Science Institute. We obtained the de-identified charts of a subgroup of patients on TRT with RCT and a matched control group based on age, sex, smoking status, diabetes diagnosis, and Charlson's Comorbidity Index. The inclusion and exclusion criteria were identical to the initial i2B2 queries. Because of a data usage agreement set forth in the institutional review board, this cohort was limited to 78 TRT users with tendon rupture and 355 matched controls with rupture (Figure 1). We reviewed patient charts and operative reports to obtain data—including magnetic resonance imaging (MRI) results, intraoperative findings, reoperation rates, and patient demographics (age and sex). We used the radiologist's reports of the MRI results and confirmed them by comparing reads to an orthopaedist's impression of images from clinic notes. We prioritized intraoperative findings if a patient had an operative report that disagreed with the radiologist's report or the orthopaedist's impression. Outcomes of interest included the rate of RCT, the rate of surgical repair versus nonoperative treatment, the rate of revision repair, and the severity of the tear. When determining the extent of the tendon tear, intraoperative findings took precedence over imaging findings.

### Statistical Analysis

A D'Agostino & Pearson test was used to test for normality of continuous data. A Welch's *t*-test was used for parametric data, the Mann-Whitney test for nonparametric data, and the chi-square test for categorical data to compare the demographic characteristics of the testosterone and control cohorts. An odds ratio (OR) with 95% CIs were calculated to quantify the risk of tendon rupture and rate of RCR/revision in each group. The chi-square test was used to compare patient characteristics and the extent of rotator cuff tendon tear. Statistical significance was set at *P*≤ .05.

## Results

### Testosterone Therapy and Upper and Lower Extremity Tendon Rupture Rate

We identified 410 patients with any tendon rupture on TRT and 14,474 patients with any tendon rupture without testosterone supplementation (Figure 1). Male patients composed the majority of the TRT cohort (381/410; 92.9%) (Table 1). Male patients on TRT had a significantly higher rate of any tendon rupture, with a rate of 3.6% compared with 1.3% for nonusers (OR, 2.88 [95% CI, 2.59-3.20]) (Table 1). This increased rupture rate persisted across age groups and racial groups. However, we did not observe a significant difference in overall tendon rupture rate between female testosterone therapy users and nonusers (1.4% vs 1%; OR, 1.36 [95% CI, 0.9-1.9]; *P* = .10). This sex discrepancy remained when examining patients with RCT specifically, with an elevated risk in male users (OR, 3.25 [95% CI, 2.88-3.66]) but not female users (OR, 1.12, [95% CI, 0.68-1.83]). The rotator cuff tendon was the most common location of rupture (77%), followed by the hamstrings (8%), the foot and ankle (4%), the Achilles tendon (3%), and the long head of biceps (1%) (Table 1).

**Table 1:** Patient Characteristics and Percentage of Tendon Ruptures per Subgroup*^*a*^*

		Total Ruptures						Rotator Cuff Tear					
		Testosterone Users		Nonusers		OR	*P*	Testosterone Users		Nonusers		OR	*P*
		(n)	%	(n)	%			(n)	%	(n)	%		
N = 1,185,017		410	3.2	14474	1.1	2.91 (2.63-3.21)	<.0001	314	2.4	9777	0.8	3.28 (2.93-3.68)	<.0001
Sex	Female	29	1.4	7505	1	1.36 (0.94-1.96)	.1004	16	0.8	5023	0.7	1.12 (0.68-1.83)	.6873
	Male	381	3.6	6969	1.3	2.88 (2.59-3.20)	<.0001	298	2.8	4754	0.9	3.25 (2.88-3.66)	<.0001
Age, years	<55	82	1.8	5292	0.7	2.66 (2.14-3.32)	<.0001	59	1.3	2512	0.3	4.04 (3.11-5.23)	<.0001
	≥55	329	4	9182	1.8	2.24 (2.00-2.51)	<.0001	250	3	7260	1.4	2.15 (1.89-2.44)	<.0001
Race	Asian	10	2.1	1048	0.9	2.33 (1.24-4.37)	.009	0	0	666	0.6	-	-
	Black or African- American	12	2.9	715	1.2	2.50 (1.40-4.45)	.002	6	1.5	427	0.7	2.07 (0.92-4.66)	.08
	White	220	3.3	6610	1.3	2.59 (2.26-2.97)	<.0001	167	2.5	4626	0.9	2.80 (2.39-3.27)	<.0001
	Others*^*b*^*	49	2.0	1590	0.03	2.77 (2.09 -3.69)	<.0001	40	1.0	1008	0.02	3.57 (2.60-4.91)	<.0001
Specific body locations	Hamstrings	22	0.23	924	0.003	3.60 (2.54-5.09)	<.0001						
	Foot and ankle^*c*^	12	0.10	417	0.001	3.13 (1.80-5.09)	<.0001						
	Achilles tendon	11	0.09	401	0.03	2.76 (1.51-5.02)	<.0001						
	Long head of the biceps	4	0.03	52	0.0003	7.73 (2.80-21.38)	<.0001						
	Others*^*d*^*	47	0.18	983	0.06	2.95 (2.58-3.36)	<.0001						

a Data are presented as OR (95% CI), unless otherwise indicated. OR, odds ratio.

b Excluding patients who did not report race or refused to answer.

c "Foot and Ankle" includes tendon ruptures of the anterior muscle group of the lower leg, the peroneal muscle group, flexor/extensor tendons of the toes, and the intrinsic tendons of the feet.

d "Others" constituted tendon ruptures in the short head of the biceps, triceps, flexor/extensor tendons at the forearm level, fingers, hips, and quadriceps.

### RCT and Repair Subanalysis

**Figure 2.**
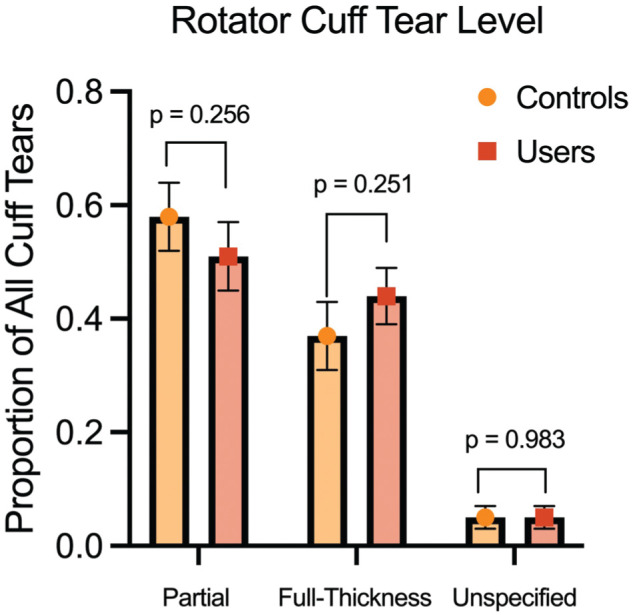
Proportion of partial-thickness, full-thickness, and unspecified tendon tears in patients with RCTs with or without TRT. MRI records and intraoperative notes in patients’ charts were used to confirm partial-thickness, full-thickness, and unspecified tears. The standard error of each proportion is depicted with error bars. MRI, magnetic resonance imaging; RCT, rotator cuff tear; TRT, testosterone replacement therapy.

We further stratified our cohort into 78 TRT patients with RCT and 355 sex, age, and propensity-score matched control patients with RCT (Table 2). The dose ranges for our male testosterone users fell within the ranges of recommended guidelines (intramuscular injection: 50-400 mg every 2-4 weeks), pellets (150-450 mg every 3-6 months), gel (10-100 mg daily), and patches (2-4 mg daily)), while the majority of female patients in our study were prescribed testosterone at male equivalent doses, which were higher than recommended guidelines for women (Supplemental Table 2).^17^ Patients on TRT with RCT received nonoperative treatment at a higher rate than controls (74.4% vs 46.7%; OR, 3.3 [95% CI, 1.91-5.72]) (Table 3). Testosterone users and nonusers had comparable rates of full-thickness and partial- thickness rotator cuff tendon tears (Figure 2). We did not observe a significant difference in revision RCR rates between groups.

**Table 2:** Demographic Comparisons of Testosterone Users and Nonusers*^*a*^*

Characteristics	Testosterone Users	Controls	*P*
Men	91	86.5	.27
Women	9	13.5	.27
Age, years	60.2 ± 9.37	60.8 ± 8.93	.07
CCI	2.12 ± 6.95	1.94 ± 1.75	.13
Comorbidities			
Dementia	2.5	0.6	.09
Obesity	7.7	3.4	.08
Morbid obesity	1.3	1.4	.93
Osteoarthritis	37	29	.17

a Data are presented as mean ± SD or %. CCI, Charlson Comorbidity Index.

**Table 3:** Rates of Operative Intervention and Revision per Subgroup*^*a*^*

			Matched Nonuser Group			
	Testosterone Users		Matched Nonusers			
	(n)	%	(n)	%	OR (95% CI)	*P*
Total	78	100	355	100	-	-
Treatment						
Nonoperative	58	74.4	166	46.7	3.30 (1.91-5.72)	<.0001
Arthroscopic RCR	20	25.6	189	53.2	-	-
Revision RCR	2	2.60	17	4.79	1.12 (0.24-5.26)	.88

a Matched groups based on age, sex, and CCI. CCI, Charlson Comorbidity Index; OR, odds ratio; RCR, rotator cuff repair.

## Discussion

This institutional study demonstrates that TRT in men is associated with significantly higher rates of tendon rupture in the upper and lower extremities, regardless of age and race. These findings are consistent with previous clinical database studies that show a strong link between testosterone supplementation and Achilles, biceps, and rotator cuff tendon injuries.^2,12,15, 19,20^ We also report an elevated risk of posterior thigh tendon (hamstring) rupture, which has not been well-characterized previously. Rotator cuff tendon tears composed the majority of injuries in our TRT cohort, and we observed a higher rate of RCT but a similar overall OR compared with previous studies.^19,20^

For RCTs, we observed that patients on TRT undergo RCR at lower rates than matched controls. This contrasts with previous database reports that testosterone users receive tendon repair surgery at a higher rate than nonusers, even in the setting of vastly higher reoperation rates.^2,19^ This is unlikely to be due to tear severity alone, as both groups had comparable rates of partial- and full-thickness tears. One reason may be that testosterone users in our cohort are offered nonsurgical treatment more aggressively due to their complication profile. Previous studies reported a >20-fold increased risk of requiring revision RCR.^19^ Although we did not observe a difference in revision rates between testosterone users and nonusers, this may be due to our smaller sample size and statistical power. Additional studies on the postoperative complication rates of patients on TRT would be most helpful in guiding the management of tendon tears in this population.

Contrary to previous studies,^2,15,19^ we did not observe elevated rupture rates in women on TRT. Most women in our study were prescribed testosterone at male equivalent doses, which were higher than recommended guidelines for women (Supplemental Table 2). Current guidelines only recommend TRT in women for the treatment of hypoactive sexual desire disorder, although there are evolving indications for its use in osteoporosis.^5^ Current guidelines recommend a low transdermal dose of approximately 300 μg daily to approximate physiologic premenopausal levels and avoid iatrogenic masculinization.^21^ This is a fraction of the 50 mg transdermal dose used for men^4^ and therefore may produce only mild deleterious effects on tendon physiology. However, in certain cases where physiologic levels of testosterone must be met, and appropriate female doses are not available, male formulations can be prescribed to women with appropriate monitoring of blood testosterone concentrations.^5^ This may explain the male equivalent doses we found in our female group. Due to the small number of female testosterone users with rupture in our study, we may be underpowered to make reasonable conclusions about the significance of our results. This group may not be representative of most female testosterone users and their risk for tendon rupture. Nevertheless, further studies are needed to clarify the relationship between sex, TRT, and tendon injury.

Basic science studies provide evidence for the direct molecular and structural consequences of exogenous testosterone that are responsible for the elevated tendon rupture rates observed in our study and previous literature. In rats that received exogenous testosterone and performed resistance training, extracellular matrix remodeling was negatively influenced at the transcriptional level.^8^ The downregulation of matrix metallopeptidase activity in the Achilles tendon of treated rats was associated with an increased likelihood of Achilles tendon injury.^11^ Testosterone administration increased tendon stiffness and failure with less elongation in rats^14^ as well as collagen dysplasia in mice,^13^ providing other mechanisms by which tendon injury can occur. In cultured human tenocytes from the supraspinatus tendon of male patients, dihydrotestosterone application led to dedifferentiation of the cell phenotype and cell proliferation, which could be associated with increased risk of tendinopathy.^6^

There are several notable limitations in this retrospective observational study. First, causation cannot be directly determined for tendon ruptures in the context of testosterone use. Although previous in vivo studies highlight mechanisms by which testosterone replacement can plausibly compromise tendon physiology, we cannot rule out confounding variables such as vigorous exercise or weightlifting, as well as the presence of patients with chronic tears presenting with acute symptoms in the TRT cohort. Second, despite the advantages of an institutional study, our sample sizes are smaller than those in previous database reports; therefore, we may be statistically underpowered to effectively examine sex effects or determine the rates of rarer outcomes such as revision RCR. Third, our study is unable to obtain granular pharmacologic details on testosterone use duration, changes to prescription, and blood levels, as many patients were initially prescribed testosterone outside of our institution or had a lack of follow-up with providers in our system concerning these prescriptions. Furthermore, we could not explore dose-dependent relationships between prescription testosterone use and tendon rupture because varying pharmacokinetics across routes of administration, differences in dosing, and patients with unknown dosages or frequency made standardization of these testosterone formulations difficult for comparison. Lastly, the results of this study reflect the prescription and treatment patterns of a single academic institution, which may not necessarily be representative of trends in other health care systems and practice settings across the United States.

## Conclusion

This single-institution retrospective study demonstrates that prescription testosterone therapy is associated with significantly increased tendon rupture rates in men but not in women, potentially reflecting sex-specific dosing practices. RCTs accounted for the majority of ruptures in patients on TRT. Although rates of full-thickness versus partial-thickness tears were comparable between groups, TRT users underwent RCR at lower rates. These findings diverge from those of previous database studies and suggest that treatment decisions in this population may be influenced by a potentially elevated risk of reinjury and reoperation. Given the rising popularity of testosterone therapy, larger multicenter studies with detailed pharmacologic data are needed to clarify dose-dependent effects, refine risk stratification, and inform tendon management strategies in this high-risk population.

## Supplemental Material

sj-docx-1-ojs-10.1177_23259671261430731 – Supplemental material for Testosterone Therapy and Associated Rates of Tendon Tear and Surgical Repair: A Retrospective Analysis Supplemental material, sj-docx-1-ojs-10.1177_23259671261430731 for Testosterone Therapy and Associated Rates of Tendon Tear and Surgical Repair: A Retrospective Analysis by Al-Hassan Dajani, Timothy P. Liu, Guillermo Araujo-Espinoza, Kevin Muy, Seth Cope, Kristofer Jones and Thomas J. Kremen in Orthopaedic Journal of Sports Medicine
