# Supplementary material for: Testosterone Therapy and Associated Rates of Tendon Tear and Surgical Repair: A Retrospective Analysis
Source: Orthop J Sports Med. 2026 Apr 20;14(4):23259671261430731. doi: 10.1177/23259671261430731 (PMC13110288; doi:10.1177/23259671261430731)
Supplement: sj-docx-1-ojs-10.1177_23259671261430731 – Supplemental material for Testosterone Therapy and Associated Rates of Tendon Tear and Surgical Repair: A Retrospective Analysis [file sj-docx-1-ojs-10.1177_23259671261430731.docx]

SUPPLEMENTARY MATERIALS

**Supplemental Table 1**: Inclusion and Exclusionary Criteria

| **Inclusionary Criteria** | Patient Demographics:   - ≥ 18 years of age - Diagnosis of Tendon Rupture (ICD-10 Code):   - · M75.1 Rotator Cuff Tear Or Rupture (2010AB-2022AB)   - · S46.00 Unspecified Injury Of Muscle(S) And Tendon(S) Of The Rotator Cuff Of Shoulder (2010AB-2022AB)   - · S46.09 Other Injury Of Muscle(S) And Tendon(S) Of The Rotator Cuff Of Shoulder (2010AB-2022AB)   - · S46.10 Unspecified Injury Of Muscle, Fascia And Tendon Of Long Head Of Biceps (2010AB-2022AB)   - · S46.19 Other Injury Of Muscle, Fascia And Tendon Of Long Head Of Biceps (2010AB-2022AB)   - · S46.20 Unspecified Injury Of Muscle, Fascia And Tendon Of Other Parts Of Biceps (2010AB-2022AB)   - · S46.29 Other Injury Of Muscle, Fascia And Tendon Of Other Parts Of Biceps (2010AB-2022AB)   - · S46.30 Unspecified Injury Of Muscle, Fascia And Tendon Of Triceps (2010AB-2022AB)   - · S46.39 Other Injury Of Muscle, Fascia And Tendon Of Triceps (2010AB-2022AB)   - · S46.80 Unspecified Injury Of Other Muscles, Fascia And Tendons At Shoulder And Upper Arm Level (2010AB-2022AB)   - · S46.89 Other Injury Of Other Muscles, Fascia And Tendons At Shoulder And Upper Arm Level (2010AB-2022AB)   - · S46.90 Unspecified Injury Of Unspecified Muscle, Fascia And Tendon At Shoulder And Upper Arm Level (2010AB-2022AB)   - · S46.99 Other Injury Of Unspecified Muscle, Fascia And Tendon At Shoulder And Upper Arm Level (2010AB-2022AB)   - · S46.90 Unspecified Injury Of Unspecified Muscle, Fascia And Tendon At Shoulder And Upper Arm Level (2010AB-2022AB)   - · S46.99 Other Injury Of Unspecified Muscle, Fascia And Tendon At Shoulder And Upper Arm Level (2010AB-2022AB)   - · S56.00 Unspecified Injury Of Flexor Muscle, Fascia And Tendon Of Thumb At Forearm Level (2010AB-2022AB)   - · S56.09 Other Injury Of Flexor Muscle, Fascia And Tendon Of Thumb At Forearm Level (2010AB-2022AB)   - · S56.10 Unspecified Injury Of Flexor Muscle, Fascia And Tendon Of Other And Unspecified Finger At Forearm Level (2010AB-2022AB)   - · S56.19 Other Injury Of Flexor Muscle, Fascia And Tendon Of Other And Unspecified Finger At Forearm Level (2010AB-2022AB)   - · S56.20 Unspecified Injury Of Other Flexor Muscle, Fascia And Tendon At Forearm Level (2010AB-2022AB)   - · S56.29 Other Injury Of Other Flexor Muscle, Fascia And Tendon At Forearm Level (2010AB-2022AB)   - · S56.30 Unspecified Injury Of Extensor Or Abductor Muscles, Fascia And Tendons Of Thumb At Forearm Level (2010AB-2022AB)   - · S56.39 Other Injury Of Extensor Or Abductor Muscles, Fascia And Tendons Of Thumb At Forearm Level (2010AB-2022AB)   - · S56.40 Unspecified Injury Of Extensor Muscle, Fascia And Tendon Of Other And Unspecified Finger At Forearm Level (2010AB-2022AB)   - · S56.49 Other Injury Of Extensor Muscle, Fascia And Tendon Of Other And Unspecified Finger At Forearm Level (2010AB-2022AB)   - · S56.50 Unspecified Injury Of Other Extensor Muscle, Fascia And Tendon At Forearm Level (2010AB-2022AB)   - · S56.59 Other Injury Of Other Extensor Muscle, Fascia And Tendon At Forearm Level (2010AB-2022AB)   - · S56.80 Unspecified Injury Of Other Muscles, Fascia And Tendons At Forearm Level (2010AB-2022AB)   - · S56.89 Other Injury Of Other Muscles, Fascia And Tendons At Forearm Level (2010AB-2022AB)   - · S56.90 Unspecified Injury Of Unspecified Muscles, Fascia And Tendons At Forearm Level (2010AB-2022AB)   - · S56.99 Other Injury Of Unspecified Muscles, Fascia And Tendons At Forearm Level (2010AB-2022AB)   - · S76.00 Unspecified Injury Of Muscle, Fascia And Tendon Of Hip (2010AB-2022AB)   - · S76.09 Other Specified Injury Of Muscle, Fascia And Tendon Of Hip (2010AB-2022AB)   - · S76.10 Unspecified Injury Of Quadriceps Muscle, Fascia And Tendon (2010AB-2022AB)   - · S76.19 Other Specified Injury Of Quadriceps Muscle, Fascia And Tendon (2010AB-2022AB)   - · S76.20 Unspecified Injury Of Adductor Muscle, Fascia And Tendon Of Thigh (2010AB-2022AB)   - · S76.29 Other Injury Of Adductor Muscle, Fascia And Tendon Of Thigh (2010AB-2022AB)   - · S76.30 Unspecified Injury Of Muscle, Fascia And Tendon Of The Posterior Muscle Group At Thigh Level (2010AB-2022AB)   - · S76.39 Other Specified Injury Of Muscle, Fascia And Tendon Of The Posterior Muscle Group At Thigh Level (2010AB-2022AB)   - · S76.80 Unspecified Injury Of Other Specified Muscles, Fascia And Tendons At Thigh Level (2010AB-2022AB)   - · S76.89 Other Injury Of Other Specified Muscles, Fascia And Tendons At Thigh Level (2010AB-2022AB)   - · S76.90 Unspecified Injury Of Unspecified Muscles, Fascia And Tendons At Thigh Level (2010AB-2022AB)   - · S76.99 Other Specified Injury Of Unspecified Muscles, Fascia And Tendons At Thigh Level (2010AB-2022AB)   - · S86.00 Unspecified Injury Of Achilles Tendon (2010AB-2022AB)   - · S86.09 Other Specified Injury Of Achilles Tendon (2010AB-2022AB)   - · S86.10 Unspecified Injury Of Other Muscle(S) And Tendon(S) Of Posterior Muscle Group At Lower Leg Level (2010AB-2022AB)   - · S86.19 Other Injury Of Other Muscle(S) And Tendon(S) Of Posterior Muscle Group At Lower Leg Level (2010AB-2022AB)   - · S86.20 Unspecified Injury Of Muscle(S) And Tendon(S) Of Anterior Muscle Group At Lower Leg Level (2010AB-2022AB)   - · S86.29 Other Injury Of Muscle(S) And Tendon(S) Of Anterior Muscle Group At Lower Leg Level (2010AB-2022AB)   - · S86.30 Unspecified Injury Of Muscle(S) And Tendon(S) Of Peroneal Muscle Group At Lower Leg Level (2010AB-2022AB)   - · S86.39 Other Injury Of Muscle(S) And Tendon(S) Of Peroneal Muscle Group At Lower Leg Level (2010AB-2022AB)   - · S86.80 Unspecified Injury Of Other Muscles And Tendons At Lower Leg Level (2010AB-2022AB)   - · S86.89 Other Injury Of Other Muscles And Tendons At Lower Leg Level (2010AB-2022AB)   - · S86.90 Unspecified Injury Of Unspecified Muscle And Tendon At Lower Leg Level (2010AB-2022AB)   - · S86.99 Other Injury Of Unspecified Muscle And Tendon At Lower Leg Level (2010AB-2022AB)   - · S96.0 Injury Of Muscle And Tendon Of Long Flexor Muscle Of Toe At Ankle And Foot Level (2010AB-2022AB)   - · S96.2 Injury Of Intrinsic Muscle And Tendon At Ankle And Foot Level (2010AB-2022AB)   - · S96.8 Injury Of Other Specified Muscles And Tendons At Ankle And Foot Level (2010AB-2022AB)   - · S96.9 Injury Of Unspecified Muscle And Tendon At Ankle And Foot Level (2010AB - Use of the following exogenous prescription testosterone or testosterone derivatives within at least 90 days of tendon rupture:   - · Androstenedione (784) (2006AC-2022AB)   - · Danazol (3102) (2006AC-2022AB)   - · Methyltestosterone (6904) (2006AC-2022AB)   - · Methyltestosterone / Pemoline / Yohimbe Preparation (1008253) (Retired)   - · Methyltestosterone / Pemoline / Yohimbine (103999) (2012AB-2022AB)   - · Nandrolone (7244) (2006AC-2022AB)   - · Oxandrolone (7779) (2006AC-2022AB)   - · Oxymetholone (7813) (2006AC-2022AB)   - · Testosterone (10379) (2006AC-2022AB)   - · Testosterone 17-Phenylpropionate / Testosterone Decanoate / Testosterone Isocaproate / Testosterone Propionate (1006908)   - · Testosterone 17-Phenylpropionate / Testosterone Isocaproate / Testosterone Propionate (1008352) |
| --- | --- |
| **Exclusionary Criteria** | Diagnoses:   - M30-M36 Systemic Connective Disorders (M30-M36) - M05 Rheumatoid Arthritis With Rheumatoid - Factor - M06 Other Rheumatoid Arthritis Mos Juvenile - Arthritis - M12 Other And Unspecified Arthropathy - L95 Vasculitis Limited To Skin, Not Elsewhere Classified - K51 Ulcerative Colitis - K50 Crohn's Disease [Regional Enteritis™] - C50 Malignant NeoplasmOf Breast   Use of the following medications within at least 90 days of tendon rupture:  **Fluoroquinolones**  · Levofloxacin (Levaquin)  · Ciprofloxacin (Cipro)  · CIprofloxacin extended-release tablets  · Moxifloxacin (Avelox),  · Ofloxacin  · Gemifloxacin (Factive)  · Delafloxacin (Baxdela).  **Corticosteroids**  · Prednisone  · Dexamethasone  · Hydrocortisone  · Methylprednisolone  **Aromatase inhibitor drug**  · Letrozole.  · Anastrozole.  · Exemestane.  **Statins**  · Atorvastatin (Lipitor®)  · Fluvastatin (Lescol. ®)  · Lovastatin (Mevacor®)  · Pravastatin (Pravachol®)  · Rosuvastatin (Crestor®)  · Simvastatin (Zocor®) |

| Supplemental Table 2: Testosterone Dosages Prescribed in Males and Females with RCT | | | | | | | | | | | | |
| --- | --- | --- | --- | --- | --- | --- | --- | --- | --- | --- | --- | --- |
| Males  (n = 71) |  |  |  |  |  |  |  |  |  |  |  |  |
|  |  | Prescription |  | Dose |  | Frequency |  | Count |  | Dose Range |  | Typical Dose  (According to Guidelines) |
| Single Form |  |  |  |  |  |  |  |  |  |  |  |  |
|  |  |  |  |  |  | Every 7 days |  | 6 |  | 75-200 MG/ML |  | 50-400 MG (every 2-4 weeks) |
|  |  | Testosterone Cypionate IM Inj in Oil |  | 200 MG/ML |  | Every 14 days |  | 3 |  |  |  |  |
|  |  |  |  |  |  | Unknown |  | 19 |  |  |  |  |
|  |  | Testosterone Cypionate IM Inj in Oil |  | 100 MG/ML |  | Unknown |  | 1 |  |  |  |  |
|  |  | Testosterone Enanthate Solution Auto-Injector |  | 75 MG/0.5ML |  | Every 7 days |  | 1 |  |  |  |  |
|  |  |  |  |  |  |  |  |  |  |  |  |  |
|  |  | Implant Pellets |  | 100 MG |  | Every 6 months |  | 1 |  | 25-100 MG Pellets |  | 150-450 mg (6–12 pellets, every 3–6 months) |
|  |  |  |  | 25 MG |  | Unknown |  | 1 |  |  |  |  |
|  |  |  |  |  |  |  |  |  |  |  |  |  |
|  |  | TD Gel |  | 100 MG |  | Every Morning |  | 1 |  | 10-100 MG Daily |  | 10-100 MG Daily |
|  |  |  |  | 50 MG/5GM (1%) |  | Every Morning |  | 4 |  |  |  |  |
|  |  |  |  | 40.5 MG/ACT(1.62%) |  | Every Morning |  | 5 |  |  |  |  |
|  |  |  |  | 30 MG/ACT |  | Every Morning |  | 3 |  |  |  |  |
|  |  |  |  | 25 MG/ACT (1%) |  | Every Morning |  | 1 |  |  |  |  |
|  |  |  |  | 20.25 MG/ACT (1.62%) |  | Every Morning |  | 13 |  |  |  |  |
|  |  |  |  | 12.5 MG/ACT (1%) |  | Every Morning |  | 4 |  |  |  |  |
|  |  |  |  | 10 MG/ACT |  | Every Morning |  | 1 |  |  |  |  |
|  |  |  |  |  |  |  |  |  |  |  |  |  |
|  |  | TD Patch |  | 4 MG/24 HR |  | Every Morning |  | 1 |  | 2-4 MG Daily |  | 2-4 MG Daily |
|  |  |  |  | 2 MG/24HR |  | Every Morning |  | 1 |  |  |  |  |
|  |  |  |  |  |  |  |  |  |  |  |  |  |
|  |  | Unknown |  | - |  | - |  | 2 |  |  |  |  |
| Multiple Forms |  |  |  |  |  |  |  |  |  |  |  |  |
|  |  | Testosterone Cypionate IM Inj in Oil |  | 200 MG/ML |  | Every 7 days |  | 2 |  |  |  |  |
|  |  | Testosterone TD Gel |  | 20.25 MG/ACT (1.62%) |  | Every Morning |  |  |  |  |  |  |
|  |  |  |  |  |  |  |  |  |  |  |  |  |
|  |  | Testosterone TD Patch |  | 4 MG/24 HR |  | Every Morning |  | 1 |  |  |  |  |
|  |  | Testosterone TD Gel |  | 40.5 MG/2.5GM (1.62%) |  | Every Morning |  |  |  |  |  |  |
| Females  (n = 7) |  |  |  |  |  |  |  |  |  |  |  |  |
| Patient |  | Prescription |  | Dose |  | Frequency |  |  |  |  |  |  |
| 1 |  | Testosterone Cypionate IM Inj in Oil |  | 200 MG/ML |  | Unknown |  |  |  |  |  |  |
| 2 |  | Testosterone TD Gel |  | 50 MG/ACT (1%) |  | Daily |  |  |  |  |  |  |
| 3 |  | Testosterone TD Gel |  | 30 MG/ACT |  | Daily |  |  |  |  |  |  |
| 4 |  | Testosterone TD Gel |  | 20.25 MG/ACT (1.62%) |  | Daily |  |  |  |  |  |  |
|  |  | Testosterone Cream |  | 0.20% |  | Daily |  |  |  |  |  |  |
| 5 |  | Testosterone (Bulk) Cream 20% |  | - |  | Unknown |  |  |  |  |  |  |
|  |  | Testosterone TD Patch |  | 4 MG/24 HR |  | Daily |  |  |  |  |  |  |
| 6 |  | Testosterone Cream 2% |  | - |  | Unknown |  |  |  |  |  |  |
| 7 |  | Esterified Estrogens & Methyltestosterone Tab |  | 1.25-2.5 MG |  | Unknown |  |  |  |  |  |  |
|  |  |  |  |  |  |  |  |  |  |  |  |  |
